# *Toxoplasma gondii* infection in slaughtered pigs and cattle in Poland: seroprevalence, molecular detection and characterization of parasites in meat

**DOI:** 10.1186/s13071-020-04106-1

**Published:** 2020-05-04

**Authors:** Jacek Sroka, Jacek Karamon, Angelina Wójcik-Fatla, Weronika Piotrowska, Jacek Dutkiewicz, Ewa Bilska-Zając, Violetta Zając, Maciej Kochanowski, Joanna Dąbrowska, Tomasz Cencek

**Affiliations:** 1 Department of Parasitology and Invasive Diseases, National Veterinary Research Institute, Puławy, Poland; 2 Department of Health Biohazards and Parasitology, Institute of Rural Health, Lublin, Poland

**Keywords:** *Toxoplasma gondii*, Pigs, Cattle, Seroprevalence, PCR, Meat, Poland

## Abstract

**Background:**

*Toxoplasma gondii* infection may pose a severe medical problem especially in a congenital form and as an acquired infection in immunocompromised persons. Raw and undercooked meat of slaughtered animals is regarded as an important source of parasite infection; however, data concerning this issue in Poland are still insufficient. The aim of this study was to estimate the prevalence of *T. gondii* infection in pigs and cattle slaughtered for human consumption in Poland using serological and molecular methods.

**Methods:**

Sera of 3111 pigs and 2411 cattle from 16 regions (voivodeships) of the country were examined for the presence of anti-*T. gondii* IgG using the direct agglutination test (DAT). Pepsin-digested samples of diaphragm and heart of seropositive animals were examined for the presence of *T. gondii* DNA (B1 gene) by nested PCR and real-time PCR, while non-digested samples were only examined by nested PCR. The B1 gene DNA samples were genotyped at 11 genetic markers using multilocus nested PCR-RFLP (Mn-PCR-RFLP) and sequencing.

**Results:**

Seropositive DAT results were found in 11.9% of pigs and 13.0% of cattle. The highest seroprevalence was found in pigs from Podkarpackie (32.6%) and in cattle from Mazowieckie (44.6%). Data analysis showed that cattle > 5–10 years-old, as well as cattle and pigs from small farms, and pigs from farms with open production systems, had higher odds of testing seropositive (*P* < 0.05). Among the examined tissue samples, positive PCR results were found in samples from 12.2% and 10.2% of seropositive pigs and cattle, respectively. Among the samples successfully genotyped by Mn-PCR-RFLP and sequenced, four samples were identified as *T. gondii* type II and one sample as type I.

**Conclusions:**

The presence of *T. gondii* antibodies in a substantial proportion of examined pigs and cattle as well as the detection of parasite DNA in their tissues highlight a potential health risk to the consumers in Poland.
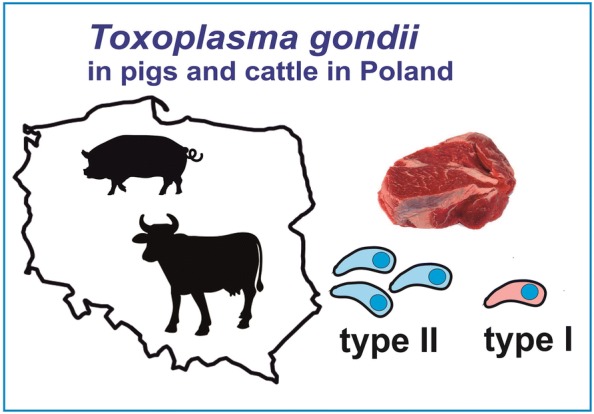

## Background

*Toxoplasma gondii* is a widespread parasite protozoan that infects warm-blooded vertebrates, including humans. Acute infection in pregnant women can be transmitted to the fetus, resulting in abortion or cerebral and ocular damage in newborns. Post-natal *T. gondii* infection can also cause ocular abnormalities, and can be life-threatening in immunocompromised individuals [1, 2]. In Poland, the seroprevalence for *T. gondii* in humans ranges between 40–60%, depending on the group of people examined [3]. According to EU law regulations, only congenital cases have recently been recorded in Poland (46 cases in 2017 and 2018) [4]. However, the overall incidence of human toxoplasmosis in Poland may still be underestimated, as other clinical forms of toxoplasmosis (i.e. lymphadenopathy, chorioretinitis and neurotoxoplasmosis) are not recorded.

Raw or undercooked meat (mainly pork) with tissue cysts containing bradyzoites, is considered a major source of human *T. gondii* infections in Europe and the USA [5]. Infection can also occur by environmentally resistant forms (oocysts) *via* contaminated water, fruit and vegetables [6]. Recently, toxoplasmosis has been ranked 4th by the WHO and the FAO among food-borne parasitic infections of global concern [7]. Moreover, the EFSA has included *T. gondii* among the most relevant biological hazards in the context of meat inspection of swine, and has pointed out that the current meat inspection procedure is unable to detect the parasite [8]. Infection with *T. gondii* in animals may also constitute a serious veterinary problem, as the parasite is associated with the occurrence of stillbirths or pathological symptoms in newborns, especially in sheep [6, 9].

Because of the significant relationship between the *T. gondii* seropositivity of pigs and sheep and the presence of live parasites in their tissues, the serological screening of these species can be especially useful for the assessment of infection risk in meat [6]. In contrast, *T. gondii* antibody detection in cattle does not strictly correspond to the presence of parasite cysts in their tissues [10, 11]. However, recent quantitative risk assessment studies showed that beef is an important source of *T. gondii* infections in the Netherlands and Italy [11–13]. The use of a combination of serology and molecular methods may serve to better assess the risk of *T. gondii* infection transmission from the consumption of meat originating from infected animals. To date, there is a lack of routine surveillance of slaughter animals for *T. gondii* infection, both at the slaughterhouse and farm levels. Therefore, it remains unknown how many animals are infected, where the endemic areas are, how large the percentage of infected animals present in the food markets is, or what role infected meat plays in overall *T. gondii* epidemiology [14].

The aim of the present study was to estimate the seroprevalence of *T. gondii* in pigs and cattle in Poland, as well as to detect *T. gondii* DNA in tissues of pigs and cattle with respect to potential threats to public health.

## Methods

### Animals and sample collection

The study was conducted as a part of the surveillance programme realized by the National Veterinary Institute in Pulawy (Poland) between 2009 and 2013, with the purpose to provide data on the contamination of foods of animal origin and the occurrence of zoonoses and epizootiologically significant animal infectious diseases in Poland. Blood and tissue samples (diaphragm and heart) were collected from pigs and cattle in abattoirs located in 16 regions (voivodeships) of Poland during slaughter and routine veterinary examinations. Samples were collected in cooperation with the Polish Veterinary Inspectorate, based on previously developed instructions. To evaluate potential risk factors associated with *T. gondii* infection, information regarding the geographical origin of animals (region of Poland), farm size, age, sex, and rearing category of animals, was collected by the Veterinary Inspectorate by means of a standardized form.

**Fig. 1:**
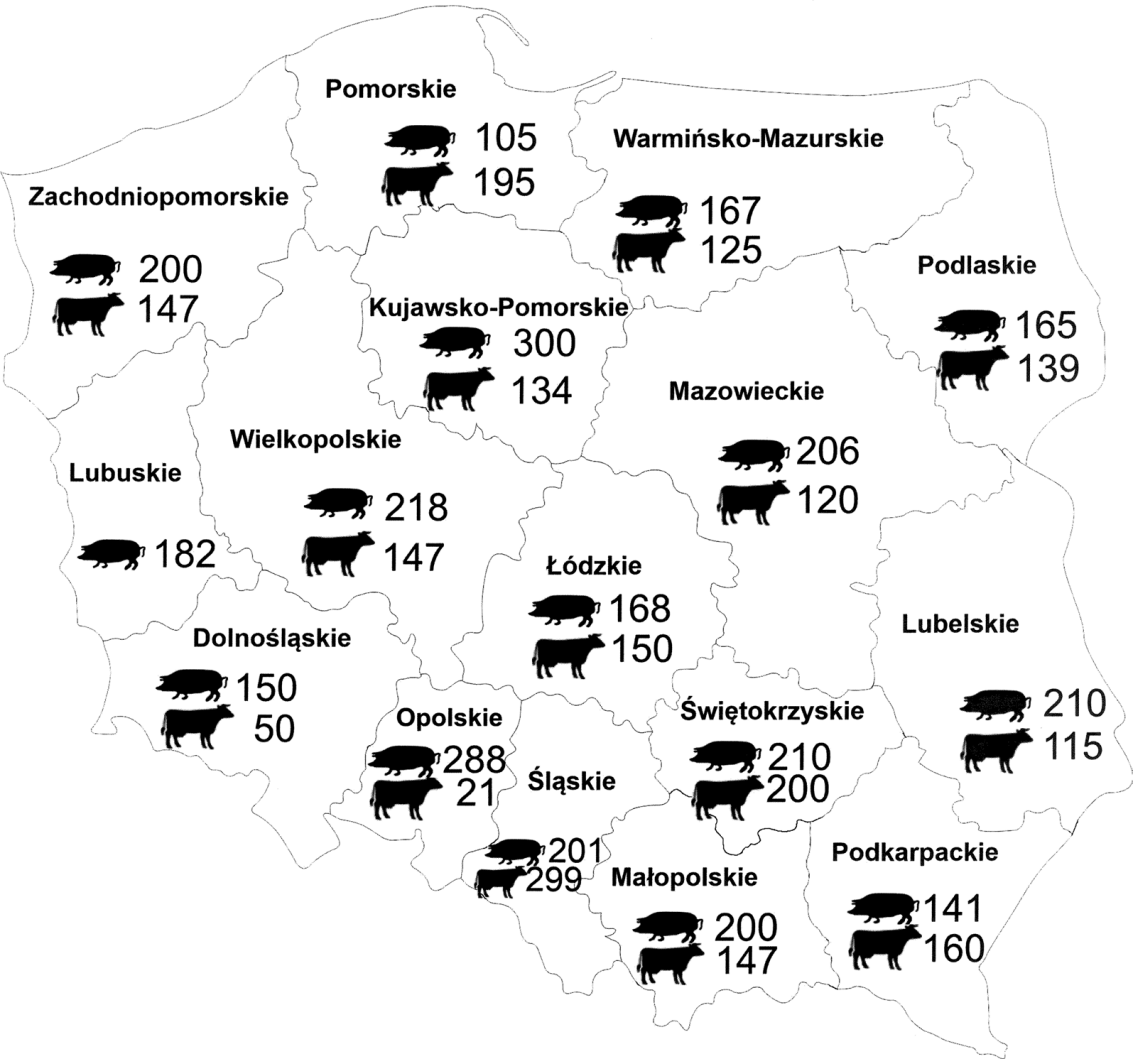
The numbers of examined pigs and cattle from individual regions (voivodeships) in Poland

In total, sera from 3111 pigs (Polish Landrace and Polish Large White breeds) and from 2411 cattle (Black-and-White Lowland breed) were collected and examined during 2009–2013 (data on the numbers of animals from individual regions of Poland are presented in Fig. 1).

Pigs came from small- or medium-scale intensive breeding farms, while cattle originated from semi-intensive or extensive rearing systems in rural areas. However, more detailed information about rearing type in relation to individual animals (cattle) was not available.

### Serological examination

The blood samples were centrifuged at 3500×*g* for 10 min and the sera were then removed and stored at − 20 °C until further analysis. Sera were examined for the presence of anti-*T. gondii* IgG using the direct agglutination test (DAT) (Toxo-Screen DA; bioMerieux, Marcy I’Etoile, France), according to the manufacturer’s instructions. Briefly, the serum samples were examined at dilutions of 1:40 and 1:4000. Samples with a titer of 1:40 or higher were considered positive. For final titer determination, positive samples were re-examined by DAT at higher dilutions, made by a factor of 3 (from 1:60 to 1:1620, and from 1:6000 to 1:162,000 for sera positive in the screening step at 1:40 and 1:4000 dilutions, respectively).

### Digestion of tissue samples and DNA extraction

Diaphragm and heart samples were stored at − 20 °C until serological analysis had been performed (2–5 days). Tissue samples of seropositive pigs and cattle were digested with pepsin solution according to the method described by Dubey and Beattie [15]. First, 50 g samples were cut and homogenized in 125 ml of 0.9% NaCl. Next, the homogenates were mixed with 250 ml of acid-pepsin solution (2.6 g of pepsin, 7 ml of HCl, and 0.9% NaCl filled up to 500 ml, pH 1.1–1.2) and digested in a shaking water bath at 37 °C for 90 min. The digested material was filtered through gauze and centrifuged at 1200×*g* for 10 min. The pellets were collected, resuspended in 35 ml of phosphate-buffered saline (PBS, pH 7.4), and centrifuged (1200× *g* for 10 min). The supernatant was removed, and the pellet was resuspended in 5 ml of 0.9% NaCl.

One hundred microliters of each suspension was used for DNA extraction using a commercial kit (QIAmp DNA Mini Kit; Qiagen, Hilden, Germany), according to the manufacturer’s instructions. DNA was also extracted from 25 mg of each homogenized tissue sample without a digestion step. All DNA samples were stored at − 20 °C until examination.

### Polymerase chain reaction (PCR)

First, all extracted DNA was examined in a nested polymerase chain reaction (PCR) for the presence of the B1 gene fragment using the method described by Grigg and Boothroyd [16]. DNA extracted from the RH *T. gondii* strain was used as a positive control and nuclease-free water was used as a negative control. The amplification products after electrophoresis were identified on an agarose gel under ultraviolet light. The PCR was carried out in a C1000 Thermal Cycler (Bio-Rad, Hercules, USA).

To increase *T. gondii* detection efficiency, DNA extracted from pepsin-digested tissue samples were additionally examined by real-time PCR according to the method by Lin et al. [17] using the commercial master mix IQ Supermix (Bio-Rad). Positive and negative controls were included as described above.

### Multiplex multilocus nested PCR-RFLP (Mn-PCR-RFLP)

Genotyping was performed using Mn-PCR-RFLP with 11 markers, including SAG1, SAG2 (5’ and 3’), altSAG2, SAG3, GRA6, BTUB, C22-8, C29-2, L358, PK1 and APICO, according to the method described by Su et al. [18].

As positive controls, GT1 (type I), PTG (type II) and CTG (type III) DNA isolates of *T. gondii* strains (kindly provided by Chunlei Su, University of Tennessee, Knoxville, USA) were used. Nuclease-free water was used as a negative control.

Each nested PCR product (5 μl) was digested with restriction endonucleases in a 20 μl volume then resolved on a 2.5% agarose gel to reveal the DNA banding pattern. The patterns of the DNA bands from each sample were compared with the genotypes deposited in ToxoDB (http://toxodb.org/toxo/).

### Sequencing

For confirmation, selected amplicons from the Mn-PCR-RFLP (52 amplicons) were sequenced by an external company (Genomed S.A., Warsaw, Poland) and sequences were analyzed using Geneious software v.11.1.4. (Geneious Co., Wellington, New Zealand) and compared with the sequences deposited in the NCBI database using BLAST.

### Statistical analysis

The numbers of animals examined in individual regions met the requirement to represent the population of these species in specific regions of the country (95% confidence level). The calculation was made using winepi.net and data regarding the size of pig and cattle populations in individual regions in Poland for 2007 (source: Statistics Poland; https://stat.gov.pl/), with an expected minimum prevalence of 1–3%, determined on the basis of the results for pigs and cattle from large, intensive farms in Poland (own data, unpublished).

To evaluate the potential risk factors, logistic regression analyses were performed using TIBCO Software Inc. (2017) Statistica (ver. 13). *P*-values < 0.05 were considered statistically significant.

Initially, univariate logistic regression analysis was used to evaluate each potential risk factor separately. The Wald test was used to evaluate the significance of individual variables. Five qualitative predictors were included in the models: region of study (voivodeship); age (cattle only; with five age groups: ≤ 1, 1–2, 2–5, 5–10 and > 10 years); sex (male and female); size of farms (small: ≤ 30 animals and large: > 30 animals); and rearing category for fattening (pigs only; closed and open pig production systems). The relationship between age and *T. gondii* infection in pigs was not analyzed due to the similar age of the majority of studied animals (5–7 months-old).

Secondly, multivariable logistic regression models were developed by including all the variables, followed by backward elimination of those with a *P*-value ≥ 0.05.

The correlation between *T. gondii* prevalence, the titre level and the results of DNA detection were assessed by Spearman’s test. Confidence intervals of the percentages of infected animals were calculated according to the method by Newcombe (http://www.vassarstats.net). The assumed level of statistical significance was 5%.

The agreement between the PCR results for pepsin-digested and non-pepsin-digested samples as well as between the PCR results for diaphragm and heart tissue samples were determined by Cohen’s kappa coefficient (κ); κ values of 0.01–0.20 indicate slight agreement, 0.21–0.40 fair, 0.41–0.60 moderate, 0.61–0.80 substantial, and 0.81–1 almost perfect agreement [19].

## Results

### Serology

Of a total of 3111 examined pigs, 369 (11.9%) were positive by DAT. Of these, high titers (≥ 1620) were detected in 2.9% of examined pigs. The highest seroprevalence was found in pigs from the Podkarpackie region (32.6%), and the lowest was found in pigs from the Pomorskie region (1.0%).

Among 2411 examined cattle, positive DAT results were found in 313 animals (13.0%). Of these, high titers (≥ 1620) were detected in 0.9% of all examined cattle. The highest percentage of seropositivity was found in the Mazowieckie region (44.6%) and lowest in the Świętokrzyskie region (3.5%) (*P* < 0.05). Among examined cattle from the Opolskie region, no positive results were found.

The distribution of seropositive pigs and cattle according to geographical origin and titer is presented in Tables 1 and 2, respectively.

**Table 1:** Results of direct agglutination test (DAT) for *Toxoplasma gondii* in pigs in Poland

Region	*n*/*N*	Seroprevalence (%)	95% CI	Titre range (%)^a^				
				40–60	180–540	1620–4000	6000	18000
Dolnośląskie	29/150	19.3*	13.8–26.4	14.0	5.3	0	0	0
Kujawsko-Pomorskie	39/300	13.0	9.7–17.3	8.0	0.7	0.7	3.3	0.3
Lubelskie	19/210	9.1	5.9–13.7	7.1	0	1.9	0	0
Lubuskie	10/182	5.5	3.0–9.8	2.2	1.1	1.6	0.5	0
Łódzkie	15/168	8.9	5.5–14.2	4.8	1.8	2.4	0	0
Małopolskie	21/200	10.5	7.0–15.5	9.0	0.5	0	1.0	0
Mazowieckie	32/206	15.5	11.2–21.1	13.6	0	1.9	0	0
Opolskie	51/288	17.7*	13.7–22.5	14.2	1.0	1.4	1.0	0
Podkarpackie	46/141	32.6*	25.4–40.7	21.3	0	11.3	0	0
Podlaskie	35/165	21.2*	15.7–28.1	10.9	6.1	1.8	2.4	0
Pomorskie	1/105	1.0	0.2–5.2	1.0	0	0	0	0
Świętokrzyskie	8/210	3.8	1.9–7.3	0.5	0	1.0	2.4	0
Śląskie	21/201	10.5	6.9–15.4	4.5	2.0	1.5	2.5	0
Warmińsko-Mazurskie	12/167	7.2	4.2–12.1	3.6	0.6	0	3.0	0
Wielkopolskie	10/218	4.6	2.5–8.2	4.1	0	0	0.5	0
Zachodniopomorskie	20/200	10.0	6.6–14.9	3.0	2.5	2.5	1.5	0.5
Total	369/3111	11.9	10.8–13.0	7.7	1.3	1.6	1.3	0.1

^a^Percent value in relation to the total number of animals examined in a given region

*Significant differences in percentages of positive results in comparison with other regions of Poland (*P* < 0.05)

*Abbreviations*: n, number of seropositive animals; N, number of animals tested; CI, confidence interval

**Table 2:** Results of direct agglutination test (DAT) for *Toxoplasma gondii* in cattle in Poland

Region	*n*/*N*	Seroprevalence (%)	95% CI	Titre range (%)^a^			
				40–60	180–540	1620–4000	≥ 6000
Dolnośląskie	5/50	10.0	4.4–21.4	8.0	2.0	0	0
Kujawsko-Pomorskie	9/134	6.7	3.6–12.3	5.2	0.7	0.7	0
Lubelskie	21/115	18.3	12.3–26.3	18.3	0	0	0
Łódzkie	13/150	8.7	5.1–14.3	5.3	2.7	0.7	0
Małopolskie	11/156	7.1	4.0–12.2	1.3	1.3	4.5	0
Mazowieckie	54/121	44.6*	36.1–53.5	30.6	14.0	0	0
Opolskie	0/21	0	0.0–15.5	0	0	0	0
Podkarpackie	13/160	8.1	4.8–13.4	4.4	3.8	0	0
Podlaskie	39/139	28.1*	21.3–36.0	20.1	5.0	2.9	0
Pomorskie	14/195	7.2	4.3–11.7	5.6	0.5	1.0	0
Świętokrzyskie	7/200	3.5	1.7–7.1	2.5	1.0	0	0
Śląskie	61/299	20.4*	16.2–25.3	17.1	2.7	0.7	0
Warmińsko-Mazurskie	34/125	27.2*	20.2–35.6	23.2	3.2	0.8	0
Wielkopolskie	25/399	6.3	4.3–9.1	5.0	0.5	0.8	0
Zachodniopomorskie	7/147	4.8	2.3–9.5	3.4	1.4	0	0
Total	313/2411	13.0	11.7–14.4	9.7	2.4	0.9	0

^a^Percent value in relation to the total number of animals examined in a given region

*Significant differences in percentages of positive results in comparison with other regions of Poland (*P* < 0.05)

*Abbreviations*: n, number of seropositive animals; N, number of animals tested; CI, confidence interval

### PCR

#### Pigs

In total, among examined tissue samples from 369 seropositive pigs, positive results by PCR were found in samples from 45 pigs (12.2%); 5 pigs were positive only by real-time PCR, 17 pigs were positive only by nested PCR, and 23 pigs were positive both by real-time PCR and nested PCR. The agreement between the nested PCR and real-time results was substantial (κ = 0.645).

However, the results largely depended on the sample processing method. Thus, the agreement between the nPCR results for pepsin-digested and non-pepsin-digested samples was only slight (κ = 0.048) and fair (κ = 0.281) for the diaphragm and heart tissue samples, respectively.

In both PCR-based methods used, more positive results were obtained for pepsin-digested samples of diaphragm than heart (19 *vs* 11 and 14 *vs* 8 samples, respectively). However, the difference between number of positive results for non-pepsin-digested samples of diaphragm and heart in the nested PCR was not significant (12 *vs* 11, respectively).

*Toxoplasma gondii* DNA (combination of PCR from digested and non-digested tissues) was more frequently detected in pigs with high antibody titers (≥ 1620; 55.6%) followed by low (40–60; 35.6%) and medium titers (180–540; 8.8%), and the association between antibody level and DNA detection was significant (*P* < 0.05).

#### Cattle

Among the examined tissue samples from 313 seropositive cattle, positive PCR results were found in samples from 32 cattle (10.2%); 19 cattle were positive only by real-time PCR, 7 cattle were positive only by nested PCR, and 6 cattle were positive both by real-time PCR and nested PCR. By nested PCR, the difference between the number of positive results for pepsin-digested and non-digested samples of diaphragm and heart was not significant (4 *vs* 6 and 2 *vs* 1 samples, respectively). However, by qPCR, more positive results were obtained for pepsin-digested samples of diaphragm than heart (16 *vs* 11 samples, respectively).

The agreement between the nested PCR and real-time PCR results was fair (κ = 0.276). No agreements (κ < 0) were recorded between the results of the nested PCR for digested and non-digested samples in particular types of samples; diaphragm and heart, and in the overall comparison of positive results for diaphragm and heart samples.

*Toxoplasma gondii* DNA (combination of PCR from digested and non-digested samples) was more frequently detected in cattle with low antibody titers (40–60; 43.7%) followed by medium titers (180–540; 31.3%) and high titers (≥ 1620; 25.0%); however, this correlation was not statistically significant (*P* > 0.1).

### Mn-PCR-RFLP and sequencing

All successfully genotyped Mn-PCR-RFLP and sequencing amplicons came only from pig samples. All amplicons were confirmed to be *T. gondii* by BLAST analysis. Five samples were amplified with eight or more markers, sufficient for probable genotype identification. Among them, 4 samples had type II alleles at almost all loci except a clonal type I allele at the Apico locus, which may correspond to the ToxoDB#3 genotype (ToxoDB). Another sample had type I alleles at all amplified loci, which may correspond to genotype ToxoDB#10 or ToxoDB#27 (ToxoDB). The results are summarized in Table 3.

**Table 3:** *Toxoplasma gondii* genotypes detected in tissues of pigs slaughtered in different regions of Poland

DNA sample ID	Locality	Results of *T. gondii* genotyping with selected genetic markers											Possible genotype (ToxoDB)^c^
		SAG1^a^	SAG2 (3’ and/or 5’)	altSAG2	SAG3	BTUB	GRA6	c22-8	c29-2	Apico	L358	PK1	
1	LS	II/III	II	II	II	II	II	II	II	I	II	na	#3
2	LS	II/III	II	II	II	II	II	II	na	I	na	II	#3
3	PK	II/III	II	II	II	II	II	na	II	I	na	na	#3
4	PK	II/III	II	na	II	II	II	na	II	I	na	na	#3
5	PK	na	na	na	II	na	II	na	na	I	na	na	nd
6	SL	na	na	na	II	na	II	na	na	I	na	na	nd
7	SL	na	na	II	na	na	na	na	na	I	na	na	nd
8	PK	na	na	na	II	na	na	na	na	na	na	na	nd
9	LD	na	na	na	na	na	na	na	na	II	na	na	nd
10	SL	na	II	na	na	na	I	na	na	na	na	na	nd
11	SL	I	I	I	I	na	I	I	na	I	na	na	#10 or #27
12	SL	na	I	na	na	na	na	na	na	I	na	na	nd
13	SL	na	I/III^b^	na	na	na	na	na	na	I	na	na	nd
14	LS	na	na	na	na	na	na	na	na	I	na	na	nd

^a^At the SAG1 locus, types II and III are indistinguishable

^b^Only the 3’ SAG2 locus was determined, types I and III were indistinguishable

^c^Available at: https://toxodb.org/toxo/

*Abbreviations*: LS, Lubuskie region; PK, Podkarpackie region; SL, Śląskie region; LD, Łódzkie region; na, product not amplified; nd, not determined

### Risk factors

Depending on the species of animal, the multivariate logistic regression analyses (*P* < 0.0001) showed a significant influence of the geographical origin (region of Poland), size of farm, age of animals, and rearing type on the serological status (positive or negative). For pigs, any multivariate logistic regression model was significant only after excluding the ‘region of study’.

#### Region of study

The multivariate logistic regression analysis showed the region of study as a risk factor for cattle (*P* < 0.0001). The analysis showed that in the Mazowieckie region (voivodeship with the highest seropositivity of 44.6%) the odds of a positive result were almost 5-fold higher than in other regions (OR: 4.83, 95% CI: 3.25–7.16).

For pigs, the univariate logistic regression analysis showed that all regions, except for Lubuskie, Świętokrzyskie and Wielkopolskie, had significantly greater odds for having a seropositive pig than the reference class in model (Pomorskie region). In Podkarpackie region, where the prevalence was the highest (32.6%), the odds of a positive result were over 50-fold greater than in the Pomorskie region (OR: 50.36, 95% CI: 6.79–373.21, *P* = 0.0001).

#### Farm size

Positive results were found more frequently among animals from small farms (≤ 30 animals) than from larger farms (> 30 animals): pigs (16.6 *vs* 6.6%); cattle (16.3 *vs* 5.5%). The multivariate logistic regression analysis confirmed that the size of the farm is another risk factor, both in pigs and cattle. The odds of a positive result were almost 3-fold (cattle; OR: 2.92, 95% CI: 1.46–5.84, *P* = 0.003) and 4-fold (pigs; OR: 3.89, 95% CI: 2.04–7.40, *P* = 0.00003) greater among animals from small farms than from larger farms (Table 4).

**Table 4:** Results of multivariate logistic regression analysis for *Toxoplasma gondii* seropositivity in pigs and cattle from Poland in relation to age, sex, farm size and rearing category

Parameter	Species		No. of animals (% seropositive)	Odds ratio	95% CI	*P*-value
Age group (years)	Cattle	≤ 1^a^	217 (9.2)	1		
		> 1–2	1241 (10.6)	1.18	0.70–2.01	0.534
		> 2–5	446 (15.2)	1.65	0.93–2.93	0.087
		> 5–10	297 (21.5)	2.42	1.33–4.40	0.004
		> 10	133 (17.3)	1.71	0.83–3.53	0.147
Sex	Pigs	Male^a^	1054 (11.3)	1		
		Female	1261 (12.5)	0.73	0.43–1.24	0.247
	Cattle	Male^a^	1128 (11.3)	1		
		Female	1009 (16.7)	1.18	0.85–1.64	0.323
Farm size	Pigs	Small (≤ 30 animals)	1885 (16.6)	3.89	2.04–7.40	< 0.0001
		Large (> 30 animals)^a^	561 (6.6)	1		
	Cattle	Small	1566 (16.3)	2.92	1.46–5.84	0.003
		Large^a^	164 (5.5)	1		
Rearing category	Fattening pigs	Open production system farms	690 (7.8)	2.67	1.42–5.02	0.002
		Closed production system farms^a^	323 (6.5)	1		

^a^Reference group in analysis

*Abbreviation*: CI, confidence interval

#### Age (cattle)

The age of the examined cattle ranged from 0.5 to 24 years-old. The mean age was 3.4 years-old (SD = 3.2). The multivariate logistic regression analysis showed that the age range > 5–10 years-old represents a risk factor (OR: 2.42, 95% CI: 1.33–4.40, *P* = 0.004) (Table 4).

#### Sex

The univariate logistic regression analysis showed significant influence of animal sex on the serological response of cattle; the odds of a positive result among females were over 1.5-fold greater than among males (OR: 1.57, 95% CI: 1.23–2.01, *P* = 0.0003); however, the significance of this dependence has not been confirmed by the multivariate logistic regression model (*P* = 0.32). No significant difference in serological response was found between female and male pigs (*P* = 0.25) (Table 4).

#### Rearing category for pig fattening

The multivariate logistic regression analysis indicated the open production system of fattening pigs as a risk factor; the odds of seropositive result in open farms were 2.5-fold greater than in closed category farms (OR: 2.67, 95% CI: 1.42–5.02, *P* = 0.002) (Table 4).

## Discussion

Recent trends in consumer habits, linked with the consumption of pork originating from free-range and organic pig farms, where the animals are exposed to *T. gondii* from the environment, may result in a higher risk of *T. gondii* infection for consumers [14]. There are still regions in Poland with small farms with traditional rearing of pigs and cattle, which have direct contact with potential sources of *T. gondii*. It has also been demonstrated that the presence of cats (frequent on Polish farms) can increase the relative risk of exposure of farm animals to the parasite by several times [20]. Having outdoor access, and animal feed stored in an area where it is possible to become contaminated with cat feces, have previously been reported as risk factors for *T. gondii* infection [21–23].

The direct agglutination test (DAT) and its commercial variant, the Toxo-Screen DA kit used in our study, are not host-species-specific [24], and are therefore useful for serological detection of the parasite, i.e. pig sera [25–27].

The total percentage of seropositive pigs found in the present study (11.9%) was slightly lower than that previously recorded in Poland, in the regions Lubelskie (up to 15.0%) [27, 28] and Wielkopolskie (13.2%) [29] and significantly lower (*P* < 0.05) compared to the results recently published by Holec et al. [30] for pigs from different regions of Poland (19.2%).

In Europe, seroprevalence in pigs varies depending on the country, farm type, and group of animals [22, 31–41], and ranges, e.g. from 4.2% in Latvia (free-ranging and intensively farmed pigs) [31] to 43.1% in Romania (backyard system) [32]. The differences in seroprevalence depend on the age of the examined animals, type of breeding, geographical region, management practices and zoohygienic status [23]. The prevalence of *T. gondii* infection in pigs is usually higher in older pigs and pigs reared outdoors than in piglets and pigs on factory farms, which was shown by studies in Serbia, Spain, Portugal and France [22, 35, 38, 40]. The benefits of intensive pig farming might be the high sanitary and technical standards that limit the possibility of contact with the parasite present in the environment, which was confirmed in the present study by the lower seroprevalence recorded for pigs from large farms compared to the results for pigs from small farms (6.6 *vs* 16.6%). Our results are in agreement with the data reported by Klun et al. [42] and Djokic et al. [40]. The seroprevalence of *T. gondii* among animal production categories may also vary. In the present study the seroprevalence in fattening pigs, bred in two types of farm production systems, closed and open, was compared. In pig farms with closed cycles, the complete exploitation process, from breeding to fattening, is carried out in the same industrial unit. In such farms, pigs can be sold at different stages, such as piglets to be fattened or as fattened pigs to be slaughtered. In open-cycle pig farms, piglets can be acquired from another pig producer for fattening and slaughter. In farms with closed production systems, the welfare requirements and sanitary safety are usually met, whereas open-cycle farms meet the requirements of biosecurity to a lesser extent. In the present study a lower seroprevalence was found in pigs from farms with closed production systems, confirming the effectiveness of preventive measures.

Data regarding the prevalence of *T. gondii* infection in cattle in Poland are scarce and mostly represented by the results of our own studies. The percentage of seropositive cattle noted in the present study (13.0%) is similar to the results of previous studies in selected regions of Poland (12.8–14.6%) [27, 43, 44] and much lower than those obtained almost 20 years ago in Lubelskie region (53.8%) [28]. A recent survey by Holec et al. [45] concerning cattle from northern Poland showed a much lower seroprevalence (3.15%). In other European countries, the seroprevalence of *T. gondii* in cattle varies [22, 36, 46–49] ranging from 7.5% in Portugal [38] to 83.3% in Spain [50].

Many studies have shown that the prevalence of *T. gondii* increases with age of examined animals; however, in cattle, a higher prevalence of *T. gondii* antibodies in younger animals was also observed, explained by the limited persistence of tissue cysts in cattle [36]. In our study, a significant correlation between age and seropositivity was observed in cattle, and seroprevalence increased with the age of the animals.

PCR (B1) performed in this study confirmed the presence of *T. gondii* DNA in tissues of 14.2% of seropositive pigs and 9.9% of seropositive cattle. The rate of DNA detection recorded in pigs in this study is much lower than that reported in Slovakia by Turčeková et al. [51], where analysis of TGR1E and B1 genes confirmed *T. gondii* in all seropositive animals (100%), and the studies performed in Italy (57.1%) [52] and in the UK (38%) [53]. Similar results (13.0%) were recorded in pigs in Ireland [41] and in Italy, where *T. gondii* DNA was detected in carcasses of 13.6% of pigs [54]. Lower percentages of *T. gondii* DNA were recently detected in retailed raw meat products from Poland (5.4%) [55] and in pigs (2.2%) and cattle (4.7%) in Switzerland [36].

In Europe, the most frequently isolated *T. gondii* strains belong to three major clonal lineages, known as genotypes I, II and III [56]. A fourth clonal genotype has been discovered in the USA [57]. Moreover, many nonclonal, atypical genotypes have been reported in South America. In contrast to the sylvatic cycle of *T. gondii*, characterized by host diversity and the occurrence of numerous atypical genotypes of the parasite, the synanthropic cycle of the parasite is mostly limited to the rearing area, and animals tend to be infected with clonal lineages [58].

In the present study, only a limited number of *T. gondii* DNA isolates from meat samples could be amplified, which may have been caused by the limited tissue sample size, small number of parasites in the original material, and random distribution of tissue cysts. The efficiency of *T. gondii* DNA detection can increase if the parasites in tissue samples are multiplied by bioassay or cell culture prior to DNA analysis, which was not possible in this study.

In this study, we assumed that the genotype could be determined when at least 8 loci are identified. On the basis of the analysis carried out in ToxoDB, four samples with type II alleles at all successfully amplified loci except one, Apico, which displayed the type I allele, may correspond to the ToxoDB #3 genotype. The same genotyping results were reported in Europe in cats [59, 60], sheep [61] and arctic foxes [62]; however, the present genotyping results seem to be different from the results of other studies in Poland, where *T. gondii* genotype III was the most prevalent in retailed raw meat products and goat milk [55, 63].

## Conclusions

The presence of *T. gondii* antibodies in a substantial proportion of examined pigs and cattle, as well as the detection of parasite DNA in their tissues indicate a potential public health risk to consumers of pork and beef in Poland.

## Data Availability

Data supporting the conclusions of this article are included within the article. The datasets used and/or analysed during the present study are available from the corresponding author upon reasonable request.

## Abbreviations

DAT: direct agglutination test
PCR: polymerase chain reaction
Mn-PCR-RFLP: multilocus nested PCR-RFLP
